# Non-Coding RNAs in Pulmonary Diseases: Comparison of Different Airway-Derived Biosamples

**DOI:** 10.3390/ijms24032006

**Published:** 2023-01-19

**Authors:** Zuzanna Stachowiak, Beata Narożna, Aleksandra Szczepankiewicz

**Affiliations:** Molecular and Cell Biology Unit, Department of Paediatric Pulmonology, Allergy and Clinical Immunology, Poznan University of Medical Sciences, 60-572 Poznan, Poland

**Keywords:** non-coding RNA, miRNA, lncRNA, circRNA, respiratory diseases, sputum, bronchoalveolar lavage, exhaled breath condensate

## Abstract

Due to their structural conservation and functional role in critical signalling pathways, non-coding RNA (ncRNA) is a promising biomarker and modulator of pathological conditions. Most research has focussed on the role of microRNAs (miRNAs), long non-coding RNAs (lncRNAs) and circular RNAs (circRNAs). These molecules have been investigated both in a cellular and an extracellular context. Sources of ncRNAs may include organ-specific body fluids. Therefore, studies on ncRNAs in respiratory diseases include those on sputum, bronchoalveolar lavage fluid (BALF) and exhaled breath condensate (EBC). It is worth identifying the limitations of these biosamples in terms of ncRNA abundance, processing and diagnostic potential. This review describes the progress in the literature on the role of ncRNAs in the pathogenesis and progression of severe respiratory diseases, including cystic fibrosis, asthma and interstitial lung disease. We showed that there is a deficit of information on lncRNAs and circRNAs in selected diseases, despite attempts to functionally bind them to miRNAs. miRNAs remain the most well-studied, but only a few investigations have been conducted on the least invasive biosample material, i.e., EBC. To summarise the studies conducted to date, we also performed a preliminary in silico analysis of the reported miRNAs, demonstrating the complexity of their role and interactions in selected respiratory diseases.

## 1. Introduction

Non-coding RNAs (ncRNAs) have been intensively studied as possible biomarkers of various human diseases, from cancers to congenital disorders. Despite a lack of coding function, they regulate multiple physiological and pathological processes. ncRNAs differ in structure, size, function and cellular location. The literature proposes a dichotomous classification of ncRNAs into housekeeping (e.g., ribosomal RNA, transfer RNA or small nuclear RNA) and regulatory (e.g., microRNA, long non-coding RNAs, circular RNA or piwi-interacting RNA) ncRNAs [1]. Due to its role in modulating gene and protein expression, regulatory ncRNAs have been studied as potential biomarkers in the diagnosis and monitoring the progression of numerous diseases.

**Figure 1:**
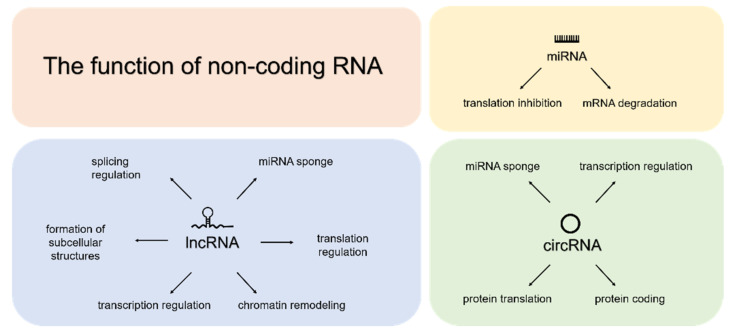
Graphical representation of the literature-described functions of three selected classes of non-coding RNAs: miRNAs, lncRNAs and circRNAs.

Many mammalian ncRNAs are expressed in a cell- and tissue-specific manner, while some are expressed ubiquitously. Highly conserved miRNAs are the most abundant short (20–25 nucleotides) ncRNAs. A mature single-stranded active molecule is formed through a stepwise process. miRNAs regulate gene transcription in the cytoplasm but are also present in extracellular fluids, where they act as signalling molecules between cells. miRNAs are responsible for the post-transcriptional regulation of up to 60% of human protein-coding genes (Figure 1). The repression of miRNA translation involves base pairing to miRNA recognition elements (MRE), usually located within the 3′untranslated region (UTR) of the target messenger RNAs (mRNAs) [2]. The research potential of miRNAs is demonstrated by miRBase evolution, as the latest release (v22) contains microRNA sequences from 271 organisms covering 48,860 mature microRNAs [3].

Less is known about long non-coding RNAs (lncRNAs), defined as RNAs longer than 200 nucleotides without protein-coding ability. They are classified based on their genomic position and divided into five subcategories: intergenic, anti-sense, sense, intronic and bi-directional [4]. lncRNAs can alter the stability and translation of cytoplasmic mRNAs and interfere with signalling pathways through cis- or trans-action. There are also reports on lncRNAs acting as miRNA sponges, thus reducing their availability to target specific mRNAs (Figure 1) [5].

Functions reported for circular RNAs (circRNA) include transcription regulation and miRNA sponging (Figure 1). circRNAs have a wide size range, from 100 to over 10,000 nucleotides, and are characterised by a covalently closed loop structure where 3′ and 5′ ends are joined and form back-splice junctions (BSJ). Due to their specific structure, circRNAs are more resistant to ribonucleases and have greater stability than linear particles. Emerging evidence indicates that circRNAs are broadly expressed in mammalian cells and show cell type- or tissue-specific expression patterns [6].

Cystic fibrosis (CF) is a common autosomal recessive disorder caused by mutations in the cystic fibrosis transmembrane conductance regulator (CFTR) gene. Characteristic, persistent high-intensity inflammation in the lungs is dominated by neutrophils that release oxidants and proteases. Patients often suffer from intermittent episodes of acute worsening of respiratory symptoms (exacerbations) caused mainly by a bacterial infection (e.g., *Staphylococcus aureus* and *P. aeruginosa*) [7,8]. Another chronic disease characterised by airway inflammation is asthma, which affects people of all ages and backgrounds. It is characterised by heterogeneous phenotypes resulting from genetic predisposition and exposure to environmental factors (e.g., pollution) [9]. Interstitial lung disease (ILD) is a highly heterogeneous condition characterised by progress in lung scarring. ILD is a collection of distinctive lung disorders classified based on clinical and pathological factors, such as idiopathic pulmonary fibrosis (IPF) and pulmonary sarcoidosis. The aetiology of individual ILD conditions can be related to infections and autoimmune diseases, as well as being idiopathic in nature [10,11].

The following review aims to overview ncRNAs identified locally in the lungs. There is a constant search for easily accessible (non-invasive) samples containing disease biomarkers. To this end, we compared different types of respiratory tract-specific biological materials in three diseases—CF, asthma and ILD—using IPF and sarcoidosis as examples. All these diseases are associated with progressive tissue remodelling and recurrent exacerbation states related to immune system hyperactivity. They are distinguished by an association or strong anticipation with a genetic background as a strong contributor to the disease susceptibility. Therefore, researchers have been analysing the influence of epigenetic factors (including ncRNAs) on their pathogenesis and progression. As a result of these considerations, diseases caused directly by environmental factors, e.g., COPD, were not included in the description. Here, we describe the functional significance of ncRNAs and their diagnostic potential based on different types of biological material.

## 2. Exhaled Breath Condensate

Exhaled breath condensate (EBC) is collected non-invasively and can be useful in studies on pulmonary disease biomarkers. EBC is obtained by cooling the exhaled air below the dew point using condenser equipment. Collection time depends on the age and state of the patient, oscillating in the range from 10 to 30 min, resulting in an obtained volume of 1–3 mL. Recommendations concerning technical aspects of the oral collection include tidal breathing and using a nose clip and a saliva trap [12].

EBC contains droplets of fluid that line the respiratory tract, water and volatile molecules from the expiratory phase. The protein profile may suggest an alveolar source of the matrix. However, the exact origin of EBC remains unspecified [13]. The high proportion of water in the collected EBC significantly influences the dilution of the collected sample, estimated to range from 20-fold up to 30,000-fold. For this reason, the main limitation of EBC is its low concentration of biomolecules. Therefore, when analysing this material, it is necessary to select sensitive assays that allow the detection of, e.g., nucleic acids. So far, studies of EBC have been focussed on small inorganic molecules (H_2_O_2_ and pH- and nitric oxide-related biomarkers), lipid mediators (8-isoprostane, leukotrienes and prostaglandins), small proteins (cytokines and chemokines) and nucleic acids (miRNA). Microbial DNA can be observed in EBC samples, which could affect diagnostic use in infectious diseases [14]. Of all the present molecules, the least is known about the non-coding RNAs in EBC (Table 1). The available studies mainly focus on cancers, where the presence and importance of miRNAs were confirmed, but the reports on other diseases are progressing. More interest in studying EBC is expected due to the presence of EVs containing locally transported nucleic acids [15,16,17].

### 2.1. Cystic Fibrosis

The first study of miRNA expression profiles in EBC was performed by Fesen et al. in CF patients. They compared the expression profiles in patients with and without *P. aeruginosa* (PA) and healthy controls to study the infection-related changes. Six miRNAs, miRNA-1247, miRNA-1276, miRNA-449c, miRNA-3170, miRNA-432-5p and miRNA-548, were over-expressed in PA samples in comparison to the other two groups. Previous reports have indicated that those six miRNAs target molecules involved in cell proliferation, inflammation (e.g., NFκβ and TNFα) or Wnt/-β-catenin pathways [18]. Our team also investigated potential differences in EV-derived miRNA expression between exacerbation and the stable stage of CF disease. We showed the presence of miR-223-3p, miR-451a, miR-27b-3p and miR-486–5p in EVs, but none of the analysed miRNAs were specific for the stage of the disease. We observed the highest increase in expression for miR-486-5p in EBC and sputum, as well as the correlation of miR-27b with lung function parameters. The latter observation is interesting because of reports on the anti-fibrotic role of this miRNA in the airways [19,20].

**Table 1:** Non-coding RNAs investigated in exhaled breath condensate (EBC) in three examples of respiratory diseases. miRNAs are classified accordingly to their reported nature as disease-differentiating or associated with the clinical parameter.

Disease	miRNA			lncRNA	circRNA	Sources
	Differentiating		Clinically Correlated/Associated			
	(↑)	(↓)				
Cystic fibrosis	No reports	No reports	miRNA-1247 miRNA-1276 miRNA-449c miRNA-3170 miRNA-432-5p miRNA-548 miR-27b-3p	No reports	No reports	miRNA: [18,19]
Asthma	No reports	miR-1248 ^###^ miR-1291 ^###^ let-7a ^###^ miR-133a miR-155 miR-570-3p ^#^	No reports	No reports	No reports	miRNA: [21,22,23,24]
Interstitial lung diseases	No reports	miR-29a	No reports	No reports	No reports	miRNA: [25,26]

^#^ specific local airway expression in comparison to peripheral; ^###^ disease-specific expression pattern (in contrast to healthy controls or different pulmonary disease).

### 2.2. Asthma

Pinkerton et al. performed the first study on the miRNA expression profile in EBC samples in asthma. Out of 39 microRNAs investigated in asthma, COPD and healthy subjects, different expression was observed for miR-1248, miR-1291 and let-7a, and these were downregulated in asthma when compared to COPD and healthy controls. Moreover, miR-133a and miR-155 levels were significantly lower in asthma than in the healthy group. This study indicated the role of EBC-derived miRNA in inflammatory processes (Th2-response) [21]. Research by Mendes et al. showed that miRNA expression in EBC could be associated with different asthma endotypes in children, defined by positive bronchodilation or an improved small airway response to treatment (salbutamol). In the first endotype, a cluster of miR-126-3p, miR-133a-3p and miR-145-5p was associated with asthma in children. Furthermore, miR-146a-5p, miR-21-5p and miR-155-5p were associated with selected asthma phenotypes [22].

Another miRNA found in EBC was miR-570-3p in the context of regulating the expression of inflammatory mediators, e.g., through targeting HuR. Roff et al. observed its downregulation in EBC samples from asthmatic patients compared to control subjects. Interestingly, changes in the expression were restricted to lung material, and in the study group, this miRNA was inversely correlated with the patient’s lung function [23]. In another study, researchers tested the effect of diet in children with asthma on miRNA expression. They suggested that high dietary acid load linked to animal-based product consumption could influence the increased expression of miR-133a-3p and downregulation of miR-155-5p in the airways of children with asthma. This observation links high intakes of animal products with possible epigenetic modifications affecting inflammatory reactions in asthma since, e.g., miR-155-5p has an anti-inflammatory role (through regulation of Th2 cytokine receptors) [24].

### 2.3. Interstitial Lung Diseases

To date, few studies have been conducted on non-coding RNAs in ILD. Ahmadzai et al. found decreased levels of miR-29a and increased expression of IFN-γ in EBC samples from patients with sarcoidosis compared to healthy controls, suggesting that miR-29a might modulate the Th1 immune response [25,26].

## 3. Sputum

The term “sputum” or “phlegm” refers to the mixture of saliva and mucus coughed up from the respiratory tract due to an existing infection or a chronic respiratory disease.

Its production occurs when respiratory tract secretions exceed the capacity of the mucociliary clearance mechanism [27]. The expectorated sputum contains secretions with cellular debris and microorganisms mainly from the lower respiratory tract, but also from the nose, mouth and pharynx. Rinsing the mouth before sample collection with clear water or saline eliminates contaminants from the oral cavity, thus avoiding misleading information about microorganisms and processes in the lower respiratory tract [28,29].

The main advantage of using sputum is its availability and non-invasive method of collection. Sputum specimens are routinely collected from adults to determine the aetiology of lower respiratory tract infections, such as pneumonia or pulmonary tuberculosis [30,31]. Due to expectoration problems, some patients require a clinician’s assistance and supportive physiotherapeutic exercises. Children tend to swallow the produced sputum rather than expectorate it, but this might be resolved with a sterile saline inhalation to loosen sputum viscosity in the lungs [32]. Sputum induction might be necessary for paediatric chronic lung inflammation disorders (such as CF) because of naturally decreased pulmonary function, respiratory muscle fatigue and/or sputum property changes [33]. Since healthy subjects do not produce sputum, it might be challenging to find the appropriate control group for the sputum studies.

Sputum is often used to study the expression profile and function of ncRNAs from the cellular pellets or supernatants collected after centrifugation. Debris material is used to investigate the cellular content, while the supernatant serves as a source of secreted molecules transported via extracellular vesicles (EVs), such as exosomes, apoptotic bodies and microvesicles [34,35]. Current research on sputum ncRNAs in selected respiratory diseases is described below, and a summary is presented in Table 2.

### 3.1. Cystic Fibrosis

There are few studies describing sputum-derived miRNAs in CF. Krause et al. showed that the miRNA cluster Mir1/Mir17-92 undergoes overexpression in CF sputum samples compared to blood neutrophils and plasma. They have also found a positive correlation between the miRNA expression in the airway material and the early exacerbation of bronchopulmonary disease [36]. This finding is particularly important, as representatives from this cluster negatively affect macrophage autophagy and impair CFTR chloride transport through macrophage membranes [37]. Our team has also investigated EV-derived miRNAs in sputum from CF paediatric patients. We found that the expression of four miRNAs (miR-223, miR-451a, miR-27b-3p and miR-486-5p) was altered in exacerbation compared to the stable stage of disease [19].

Mucus in CF is a significant barrier in the delivery of new therapies due to its increased density and viscosity. One of the investigated therapeutic approaches involves the direct delivery of nano-encapsulated siRNA to the airways [38]. siRNA silences target genes (e.g., the alpha subunit of the epithelial sodium channel, *αENaC chain*) and subsequently improves the clinical condition of patients [39]. Unfortunately, the sputum properties are influenced by neutrophil infiltration, neutrophil-derived DNA and microorganism colonisation, which impacts the uptake of nanoparticles [40]. Recent studies of sputum in CF have considered the specificity of this biological material when searching for structural modifications of nanoparticles affecting the transport efficiency and assisting the entrapped siRNA diffusion through the mucus [41].

### 3.2. Asthma

The research on sputum in asthma was focussed on identifying the molecules responsible for the severity and progression of the disease. This gives an insight into the asthma endotypes, phenotypes and pharmacogenetic mechanisms [28,29,42,43].

miRNA profiling in sputum was performed by Maes et al. to investigate the differences in the severity of asthma and inflammatory phenotypes. Upregulation of miR-629-3p, miR-223-3p and miR-142-3p was significantly associated with severe neutrophilic asthma, suggesting their potential to predict disease severity. For example, transfection with the miR-629-3p mimic induced the expression of mRNA and the protein of interleukin 8 (*IL-8*), involved in the neutrophil-mediated immune response [44] in airway epithelial cell culture [45].

This finding is in line with a previous study where a sputum miRNA module (“*nely*”) was identified: miR-223-3p, let-7a-5p, let-7d-5p, let-7 g-5p, let-7i-5p, miR-15b-5p, miR-23a-3p, miR-25-3p, miR-150-5p, miR-181a-5p, miR-191-5p and miR-342. This module correlated with clinical parameters such as absolute neutrophil and lymphocyte sputum counts and showed enrichment for phenotypic traits associated with severe asthma [46]. In another study, miR-199a-5p overexpression was associated with the neutrophilic phenotype of asthma peripherally as well as in the lungs, and was strongly related to impaired pulmonary function [47]. A different study on glucocorticoid responsiveness mechanisms indicated miR-9 overexpression in the sputum of patients with neutrophilic asthma compared to the eosinophilic endotype [48].

Studies on miRNAs’ role in eosinophilic asthma have shown that miR-221-3p levels in sputum and plasma were significantly lower in patients with asthma compared to healthy controls. and were negatively correlated with airway eosinophilic inflammation. Moreover, an anti-inflammatory chemokine *CXCL17* gene was indicated as a potential target for miR-221-3p. Interestingly, increased CXCL17 levels could suppress the expression of pro-inflammatory chemokines involved in eosinophilia, suggesting the protecting role of miR-221-3p downregulation [49].

In the study on asthma-chronic obstructive pulmonary disease (COPD) overlap syndrome (ACOS), the biomarker function of miR-145 and miR-338, compared to samples from patients with asthma and COPD, was not confirmed. However, both these miRNAs were overexpressed in sputum compared to in blood from asthma patients [50].

Studies on inflammation in allergic asthma suggest a potential local airway involvement of downregulated miR-155 [51]. Monitoring miRNA expression in the sputum can be used to study the mechanisms and efficacy of anti-allergy therapy. For example, Jakwerth et al. observed the increased expression of miR-3935 in sputum-derived immune cells after allergen-specific immunotherapy. Furthermore, they indicated a negative regulatory relationship between this miRNA and its predicted target gene, the *PTGER3* receptor. It is interesting since PTGER3 activation by prostaglandin E2 (PGE2) has a bronchodilatory (on smooth muscle cells) and immunosuppressive effect (influences, e.g., macrophages or B cells) [52].

Previous studies that attempted to link lncRNAs functionally with miRNAs showed that lncRNA-NEAT1 could act as a sponge for miR-128 in the airways of asthmatic children. Elevated sputum levels of lncRNA-NEAT1 were associated with an enhanced inflammatory reaction, while miR-128 showed the opposite effect [53]. Interestingly, similar correlations between this lncRNA and miR-124 were also observed in the plasma of asthmatic patients [54].

### 3.3. Interstitial Lung Diseases (including ChILD, Sarcoidosis and IPF)

Sputum has also been proposed as a valuable, non-invasive method for the assessment of ILD [55]. Ongoing studies seek to clarify the basis of ILD, including idiopathic pulmonary fibrosis (IPF). Njock et al. screened sputum-derived exosomes for differentially regulated miRNAs and found that three molecules: miR-142-3p, miR-33a-5p (upregulated) and let-7d-5p (downregulated) were altered in IPF samples. This preliminary analysis suggested their potential role as disease biomarkers [56]. Guiot et al. also investigated the exosomal transport of miRNA in the sputum of IPF patients and found specific upregulation of miR-142-3p in IPF compared to healthy controls, correlating with the percentage of sputum-derived macrophages. This observation suggested that sputum macrophages may be a source of elevated exosomal miRNA that regulated the expression of *TGFβ-R1*, involved in fibrosis [57].

**Table 2:** Non-coding RNAs investigated in sputum in three examples of respiratory diseases. miRNAs are classified accordingly to their reported nature as disease-differentiating or associated with the clinical parameter.

Disease	miRNA			lncRNA	circRNA	Sources
	Differentiating		Clinically Correlated/Associated			
	(↑)	(↓)				
Cystic fibrosis	miR-17 ^#^ miR-18a ^#^ miR-19a ^#^ miR-20a ^#^ miR-19b-1 ^#^ miR-92a ^#^	No reports	miR-223 miR-451a miR-27b-3p miR-486-5p	No reports	No reports	miRNA: [19,36]
Asthma	miR-629-3p miR-223-3p miR-142-3p miR-9 ^##^ miR-145 ^#^ miR-338 ^#^	miR-221-3p miR-155	*Nely* module: (let-7a-5p let-7d-5p let-7g-5p let-7i-5p miR-15b-5p miR-23a-3p miR-25-3p miR-150-5p miR-181a-5p miR-191-5p miR-223-3p miR-342) miR-199a-5p miR-3935	lncRNA-NEAT1	No reports	miRNA: [45,46,47,48,49,50,51,52] lncRNA: [53]
Interstitial lung diseases	miR-142-3p miR-33a-5p	let-7d-5p	No reports	No reports	No reports	miRNA: [56,57]

^#^ specific local airway expression in comparison to peripheral; ^##^ endotype specific expression.

## 4. Bronchoalveolar Lavage and Bronchial Brushings

Bronchoalveolar lavage fluid (BALF) is collected during the bronchoscopy. BAL (bronchoalveolar lavage) procedure involves introducing and aspirating a sterile saline solution from a sub-segment of the lung. Analysis of cellular and non-cellular components of the fluid can potentially provide valuable information about airway inflammation [58].

This method allows the study of the cellular (genetic and cytological) and environmental (microbiological) conditions prevailing in the lower respiratory airways that reflect lung diseases that occur at the alveolar level. The data obtained from BAL can be compared to results from a bronchial biopsy. Still, the second method is less safe, more invasive and collects a smaller quantity of the material. Importantly, the BAL sample extends beyond the bronchi and can be analysed for white and red cells, fungi, mycobacteria, bacteria or exosomes. Exosomes in BAL are released mainly by epithelial cells and macrophages. It is also possible to detect viral infections (e.g., CMV) with a combination of culture and cytological examination of epithelial cells [59,60].

A bronchoscopy also allows for the collection of bronchial brushing (BB). Sampling occurs via the gentle wedging of a sheathed cytology brush in the airways. Obtained airway epithelial cells can be used for immediate analyses or primary bronchial epithelial cell cultures. Material acquired with this method is also used to study pathological processes in the respiratory tract, including the expression of non-coding RNAs such as miRNA and circRNA [61,62]. Current research on ncRNA in selected respiratory diseases is presented below and summarised in Table 3. Both materials can be collected from adults as well as paediatric patients. Again, since BAL and BB are not performed on healthy individuals, it is difficult to obtain reference values [63].

### 4.1. Cystic Fibrosis

Previous studies have indicated an inhibitory effect of the genetic modifier TGF-β on *CFTR;* therefore, Kabir et al. investigated whether TGF-β-based inhibition of *CFTR* expression could be affected by miRNAs isolated from BAL-derived exosomes of CF patients. In silico analysis identified miR-145, miR-101 and miR-494 as microRNAs targeting the 3′UTR region of *CFTR*. Elevated levels of both miR-145 and TGF-β (and their signalling pathway genes) in CF were confirmed in BAL, and a functional assay verified that TGF-β stimulation upregulated miR-145 transcription in airway epithelia. miR-145 antagonists in the cell model led to *CTFR* upregulation and increased the corrector (lumacaftor) efficiency [64,65].

A study on hypoxia with BAL from upper and lower lung lobes showed differential expression of miR-451a and miR-663 [66]. Importantly, BAL, as a disease-specific fluid, reflects the local state of the airways and can be used in in vitro models to study the function of miRNAs under conditions resembling those of the respiratory tract, e.g., the effect of miR-17 on *IL8* levels or miR-143-5p on *CFTR* [67,68].

With regard to non-coding RNAs, much more studies have focussed on epithelial cells collected by bronchial brushings. Weldon et al. found miR-31 to be significantly downregulated in CF compared with non-CF samples. This miRNA is highly expressed in human non-CF airway epithelial cells, with decreased expression in diseases such as cancer, coronary artery disease or lupus [69]. Another study reported downregulated expression of miR-126, which could lead to TOM1 upregulation. Interestingly, through direct targeting of the *TOM1* 3′UTR region by miR-126, NF-κβ-regulated IL8 secretion may be enhanced. Therefore, this miRNA may regulate the anti-inflammatory reactions in CF lungs [70].

The regulatory role of miRNA in IL-8-mediated inflammation in CF was observed in a study by Bhattacharyya et al., who found significantly elevated miR-155 levels in CF bronchial brushing neutrophils. As overexpression of miR-155 may inhibit the expression of SHIP1, a protein that destabilises IL-8 by deactivating the PI3K/Akt signalling pathway, this miRNA may promote the pro-inflammatory CF phenotype by stabilising IL-8 mRNA [71].

Another study focussed on miRNAs that are computationally predicted to regulate CFTR, and found that four miRNAs were upregulated (miR-101, miR-145, miR-223 and miR-494) and three were downregulated (miR-31, miR-331-3p and miR-362-5p) in BB-derived epithelial cells (with at least one ΔF508 *CFTR* allele). Further experiments confirmed overexpression of miR-145 and miR-223, and a negative correlation between miR-494 and *CFTR* expression. According to the authors, *P. aeruginosa* colonisation and inflammation in ΔF508 *CFTR* patients may affect miRNA expression and contribute to CFTR dysregulation [72]. Increased levels of miR-145 together with miR-221 and miR-494 were also observed in the study on *ATF6* targeting miRNAs in *ΔF508-CFTR* samples. Authors hypothesised that the downregulation of ATF6 may influence effective protein folding in the endoplasmic reticulum (ER) through unfolded protein response networks [73]. Furthermore, De Santi et al. investigated the potential of upregulated miR-143-5p in bronchial brushings from CF patients, and found that this miRNA inhibits *CFTR* by binding to its miRNA recognition elements (MRE) [68]. They also demonstrated that inhibition of miR-145-5p and miR-223-3p binding sites in combination with correctors improved ion transport by ΔF508-CFTR [74].

McKiernan et al. performed the first study investigating the lncRNA expression profile in the BB cells of CF patients and found 1063 differentially expressed lncRNAs, including XIST, TLR8-as1, MALAT1 and HOTAIR. However, validation with a quantitative real-time polymerase chain reaction (qRT-PCR) did not confirm significant differences for selected ncRNAs, but showed an inverse level of *TLR8* mRNA relative to the non-coding TLR8-AS1 [75].

### 4.2. Asthma

In adult patients with mild non-symptomatic asthma, Levänen et al. observed differential expression of 24 exosomal miRNAs in BALF compared to healthy control subjects. A subset of 16 miRNAs best distinguished the classified study groups (but only seven miRNAs: let-7a, miR-21, miR-658, miR-26a, miR-99a, miR-200c and miR-1268 were further validated). Interestingly, the suggested function of the let-7 family is the inhibition of pro-inflammatory IL-13 (Th2 cell responses), while the miRNA-200 family potentially regulates epithelial-mesenchymal transition. Therefore, their downregulation in asthma may lead to impaired inhibition of inflammation [76]. Subsequent studies confirmed lower expression of miR-200b and miR-200c in BAL of asthmatic patients, and their reduced expression was specific to moderate as well as severe asthma. Moreover, both miR-200b and miR-200c target the 3’UTR of *IL-33*, which encodes proteins overexpressed in asthma and released during tissue damage and activation of cells involved in allergic inflammation, such as eosinophils or macrophages [77]. In infants with asthma, Zhang et al. investigated the expression of let-7 representatives (7a, 7b and 7c) and reported upregulation of these miRNAs in BALF, in contrast to the results obtained for the let-7 family by Levänen et al. [78]. However, study group classification seems unclear, as the article generally refers to the Global Initiative for Asthma (GINA) guidelines without indicating specific criteria or lung function tests that were used for diagnosing asthma in infants. The inconsistency between the reports could derive from the different study groups, since Zhang et al. included patients under five years of age (which is the standard age of diagnosis) and compared asthma with patients suffering from airway foreign bodies, not healthy controls. Another study with older children, but with the same clinical comparison as Zhang et al., reported elevated expression of miR-26a, miR-146a and miR-31 in BALF of asthmatic patients. These miRNAs have also been shown to be involved in remodelling and airway inflammatory response [79].

In severe asthma (SA), the total mature miRNA load in EVs from BAL samples was decreased in comparison to healthy controls. Additionally, around 23% of detected microRNAs were downregulated in SA subjects. An in silico model showed that reduced expression of selected microRNAs in SA was predicted to increase the expression of genes involved in inflammation and remodelling. Researchers compiled selected miRNAs with clinical data and showed that miR-625-3p, miR-202-5p, miR-202-3p, miR-568 and miR-151a-5p correlated with a reduction in the predicted FEV1%. In addition, miR-615-3p, miR-10b-5p and miR-151a-3p were inversely correlated with eosinophil counts, whereas miR-224-5p, miR-581, miR-151a-5p and miR-9-5p inversely correlated with the neutrophil numbers [80].

In acute asthma, the differential expression of three miRNAs was observed: miR-150, miR-152 and miR-375, which bind *TLR7* in macrophages from BAL and were associated with an impaired response to viral infections (IFN pathway) in severe asthma [81].

In asthma, more investigations on miRNA expression were performed in cells harvested by bronchial brushings. Jardim et al. investigated differential miRNA expression profiles in BB from asthmatic patients without endotype-dependent sample separation. They found 66 miRNAs and validated three that were upregulated, let-7f, miR-487b and miR-181c, and one that was downregulated, miR-203. Through in silico analysis, researchers assigned *AQP4*, which was upregulated in asthma, as a potential target for miR-203 which is assumed to influence the movement of osmotically driven water and tissue swelling [82]. Solberg et al. found 217 differentially expressed miRNAs in steroid-naive subjects with asthma compared to healthy controls. They validated 22 miRNAs (including miR-34/449 family members) and showed that downregulated members of this family (miR-34c-5p, miR-34c-5p, miR-449a and miR-449b-5p) may be repressed by IL-13 exposure in in vitro experiments. Authors suggested that the IL-13/miR-449 interaction may act by a possible NOTCH1 pathway, triggering airway mucous metaplasia and inhibiting alveolar development [83].

The link between the expression of miRNAs and specific inflammatory cells was also investigated in asthma endotypes. Two miRNAs downregulated in asthma, miR-181b-5p and miR-221-3p, were isolated from BB and found to be inversely correlated with airway eosinophilia. Huo et al. showed that miR-181b-5p targets *SPP1* and regulates the expression of pro-inflammatory cytokines, IL-1B and CCL1 [84], whereas miR-221-3p may inhibit inflammatory cytokine expression in type 2-high asthma by upregulating the chemokine CXCL17. This observation was also confirmed in the BB and serum of asthmatic patients [49]. Another miRNA with a potential protective role in asthma is miR-218-5p. Liang et al. showed its negative correlation with eosinophils in induced sputum and BB and also showed that it targeted pro-eosinophilic *CTNND2*. The authors also suggested the miR-218-5p/CTNND2 pathway’s role in regulating chemokine CCL26 in the airways [85]. Recent research on eosinophilia in asthma reported downregulated expression of miR-30a-3p in BB of patients. Wu et al. observed that its expression was negatively correlated with sputum and airway eosinophil counts and suggested the enhancement of *RUNX2* (miR-30a-3p target) that regulates *HMGB1* expression. HMGB1 was reported to enhance the survival of eosinophils and served as their chemoattractant. RUNX2 can also bind SPP1, previously described as a pro-inflammatory molecule [86]. Regarding neutrophilic inflammation in asthma, Kivihall et al. observed the downregulation of miR-146a in asthmatic BB cells. Its reduced expression negatively correlated with neutrophil cell count in the airways and was suggested to lead to the increased neutrophil-attracting chemokines IL-8 and CXCL1 [87].

Moreover, using in vitro model, researchers observed upregulation of miR-221 in lung injury BB cells from asthmatic patients. Authors suggested its potential to inhibit the proliferation of bronchial epithelial cells via targeting the *SIRT1* gene [88].

Nicodemus-Johnson et al. observed elevated miR-148b and miR-152 in BB from subjects with a family history of asthma (diagnosed in the mother). The functional analysis of these miRNAs suggested regulation of *HLA-G* by translation inhibition. Researchers hypothesised that the pathogenesis of asthma might differ among offspring of asthmatic versus non-asthmatic mothers due to miRNA/*HLA-G* regulation [89]. MiRNAs were also analysed in type 2-low and type 2-high asthma endotypes with regard to cytokine expression. Rider et al. observed that miR-206 regulated airway IL-25 and TSLP expression by targeting the CD39/extracellular ATP axis that could contribute to type 2-high asthma and also account for the therapeutic potential of the indicated microRNA [90].

So far, no reports concerning BALF-derived lncRNA have been reported to date. With regard to circular RNAs in BALF, Wang et al. found 160 differentially expressed circRNAs in BALF of allergic asthma patients compared to the patients with foreign body aspiration. circZNF652 (upregulated in asthmatic children) showed 93% homology with mice. Studies in the animal model suggested that this circRNA may bind miR-452-5p, and the researchers described the potential role of circZNF652 in inducing the expression of Janus Kinase 2 (JAK2) and activation of JAK2/STAT6 signalling, resulting in goblet cell metaplasia [91].

LncRNA profiles in BB airway endothelial cells were studied by Liu et al., who used expression data from the GSE67472 dataset (NCBI GEO) and found 159 lncRNAs differentially expressed in asthma versus healthy controls. One of them, lncRNA ZNF667-AS1 (lncRNA MORT), with previously proven anti-inflammatory potential in other diseases, was downregulated in asthma. In silico analysis showed that key lncRNAs and mRNAs were involved in core signalling pathways, such as activation of PPAR signalling and eosinophil apoptosis, leading to the development and progression of asthma [92]. A similar analysis method was used by Chen et al., who investigated circRNA-miRNA-mRNA networks in asthma BB cells based on available expression datasets. Starting from identifying hub genes, the researchers established functionally related miRNAs and extended the analysis to circRNAs with regulatory potential. The final circRNA-miRNA-mRNA network included three circRNAs (hsa_circ_0001585, hsa_circ_0078031 and hsa_circ_0000552), twenty-seven miRNAs and twelve mRNAs. Although some of these mRNAs and miRNAs have been reported in asthma, their function requires further investigation [62].

**Table 3:** Non-coding RNAs investigated in bronchoalveolar lavage fluid (BALF) and bronchial brushing (BB) in three examples of respiratory diseases. miRNAs are classified accordingly to their reported nature as disease-differentiating or associated with the clinical parameter.

Disease	miRNA			lncRNA	circRNA	Sources
	Differentiating		Clinically Correlated/Associated			
	(↑)	(↓)				
Cystic fibrosis	miR-145 miR-155 miR-101 miR-223 miR-494 miR-221 miR-143-5p	miR-31 miR-126 miR-331-3p miR-362-5p let-7b miR-17 miR-203	miR-451a miR-663	XIST TLR8-as1 MALAT1 HOTAIR	No reports	miRNA: [64,65,66,67,68,69,70,71,72,73,74] lncRNA: [75]
Asthma	miR-1268 miR-658 let-7a ^###^ let-7b ^###^ let-7c ^###^ miR-26a ^###^ miR146a ^###^ miR-31 ^###^ miR-150 miR-152 miR-375 let-7f miR-487b miR-181c miR-1246 miR-663a miR-1248 miR-1915-3p miR-665 miR-1268a miR-1207-5p miR-221 miR-206 ^##^	let-7a miR-21 miR-26a miR-99a miR-200c miR-200b miR-203 miR-107 miR-7c miR-31-5p miR-99a-5p miR-29a-3p miR-449a miR-106b-5p miR-19b-3p miR-27b-3p miR-449b-5p miR-200a-3p miR-27a-3p miR-29c-3p miR-34b-5p miR-34c-5p miR-181b-5p miR-218-5p miR-30a-3p miR-146a miR-206	miR-625-3p miR-202-5p miR-202-3p miR-568 miR-151a-5p miR-615-3p miR-10b-5p miR-151a-3p miR-224-5p miR-581 miR-9-5p miR-148b	ZNF667-AS1 (lncRNA MORT)	circZNF652 hsa_circ_0001585 hsa_circ_0078031 hsa_circ_0000552	miRNA: [49,76,77,78,79,80,81,82,83,84,85,86,87,88,89,90] lncRNA: [92] circRNA: [62,91]
Interstitial lung diseases	miR-150 miR-146a miR-192 miR-221 miR-24 ^#^ miR-27a ^#^ miR-34a ^#^ miR-125b ^#^ miR-146b ^#^ miR-155 ^#^ miR-22-3p miR-320a-3p miR-320b miR-24-3p	miR-202 miR-204 miR-222 miR-15b miR-130b ^#^ miR-150 ^#^ miR-30a-5p miR-375-3p ^###^ miR-200a-3p miR-200b-3p miR-141-3p miR-423-5p miR-29a miR-185	miR-155 let-7c miR-146a miR-150	No reports	No reports	miRNA: [93,94,95,96,97,98,99,100]

^#^ specific local airway expression in comparison to peripheral; ^##^ endotype specific expression; ^###^ disease-specific expression pattern (in contrast to healthy controls or different pulmonary disease).

### 4.3. Interstitial Lung Diseases

Researchers investigated the expression of 25 miRNAs with possible involvement in the regulation of inflammatory response in sarcoidosis. They showed that miR-150 and miR-146a were upregulated, while miR-202, miR-204 and miR-222 were downregulated in sarcoidosis versus the control. Interestingly, after a 2-year follow-up, only miR-155 and let-7c demonstrated higher expression in patients with progressing disease (compared to the regression group) [93]. Kiszałkiewicz et al. investigated the expression of let-7f, miR-15b, miR-16, miR-20a, miR-27b, miR-128a, miR-130a, miR-192, miR-221 and miR-222 in BALF supernatants collected from patients with pulmonary sarcoidosis. Those miRNAs were reported to target VEGF, TFG-β, SMADs and HIF-1A involved in sarcoidosis pathogenesis. However, only miR-15b, miR-192 and miR-221 showed altered expression in BALF as compared to healthy controls. The expression of miR-16 and miR-128a overlapped in the lungs and blood lymphocytes of sarcoidosis patients and showed higher expression in patients than in controls [94].

Another study confirmed the presence of four miRNAs, miR-21, miR-26a, miR-146a and miR-150, in BALF-derived extracellular vesicles (EVs) from adult sarcoidosis patients. Furthermore, miR-146a and miR-150 were upregulated in patients in stage II of the disease (CXR-II) and negatively correlated with selected lung function parameters [95]. In the study on acute sarcoidosis, researchers compared miRNA profiles in the regulatory T cells (Tregs) derived from blood and BALF. miR-24, miR-27a, miR-34a, miR-125b, miR-146b and miR-155 were upregulated, and miR-130b and miR-150 were downregulated in the airway material. Interestingly, none of these miRNAs differentiated sarcoidosis samples from controls [96].

Concerning IPF, Liu et al. found altered expression of 68 miRNAs in BALF exosomes of elderly patients when compared to healthy controls. Moreover, the researchers showed that downregulated miR-30a-5p in IPF may decrease TAB3 protein expression and influence chronic inflammatory responses and tissue remodelling. This observation is consistent with the existing evidence of the anti-fibrotic role of miR-30a-5p in liver fibrosis [97]. A recent study by Kaur et al. also studied the miRNA profile in BALF-derived exosomes of IPF patients. They reported that miR-375-3p, miR-200a-3p, miR-200b-3p, miR-141-3p and miR-423-5p were significantly downregulated, while miR-22-3p, miR-320a-3p, miR-320b and miR-24-3p were upregulated in the BALF of IPF patients in comparison to healthy controls [98].

In another study, researchers found a decreased expression of miR-29a and miR-185 in IPF patients when compared to healthy subjects. Tsitoura et al. discovered a negative correlation of miR-29a with type I collagen and proposed a potential pathway linking miR-185 and AKT protein [99]. The same research group showed an equivalent expression pattern of these two miRNAs in BAL from patients with IPF and lung cancer. A significant difference was observed in the levels of collagen 1, a potential target of miR-29a, in IPF but not in lung cancer samples. These findings may contribute to a better understanding of fibrotic processes in IPF [100].

So far, there are no reports on non-coding RNAs isolated from BB in interstitial lung diseases.

## 5. In Silico Potential of Non-Coding RNAs in Different Lung Diseases

Based on the review of the existing literature on non-coding RNAs in airway diseases, it is clear that miRNA expression and function studies are more advanced than for other classes of ncRNAs. Disproportion is also noted in the type of biological material studied, since the least explored is EBC (Table 1, Table 2 and Table 3). These inequalities stem from the nature of described samples, different stability of ncRNA types, lower abundance and poorer bioinformatic tolls for lncRNAs and circRNAs. Looking at the information provided by miRNA studies, extending the research focus to other classes of non-coding RNAs is recommended.

To investigate the miRNA biomarker potential from previous studies, we conducted a preliminary in silico analysis of the presented miRNA expression signatures. This way, miRNA interaction specificity in selected respiratory diseases was tested. Panels of miRNAs that differentiated between airway diseases and reference groups were examined using the mirDIP database (Version 5.2.2.1), with score classes high–very high and the number of sources (assigning miRNAs to its specific target genes) set to >10. With these criteria, the algorithm gave targets for all investigated miRNAs (except for miR-19b-1). Next, we performed functional annotation for individual sets of target genes with DAVID (version 2021). We included KEGG non-cancer pathways from the annotation tool according to the criteria: adjusted Benjamini–Hochberg *p* < 0.05, false discovery rate (FDR) < 0.05 and EASE < 0.05. The pathways were ranked by the lowest *p*-value and the highest fold enrichment.

**Figure 2:**
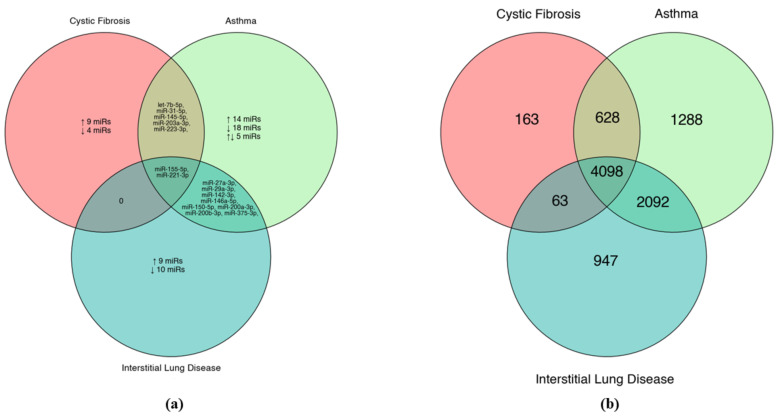
Venn diagrams of (**a**) disease-differentiating miRNAs studied in selected biomaterials, with (↑/↓) indicating disease direction of expression and (**b**) predicted gene targets of miRNAs differentiating selected diseases, acquired through miRDip database. Developed with RStudio.

**Figure 3:**
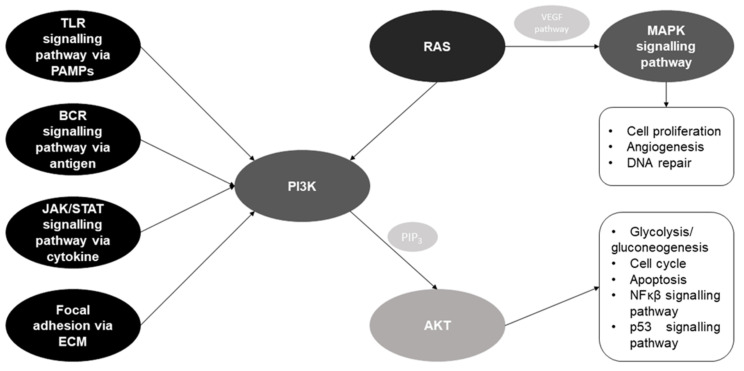
A general schematic diagram illustrating the directional links between signalling pathways containing target genes for miRNAs differentiating selected respiratory diseases. Black fields represent pathways directly or indirectly impacting the PI3K or MAPK pathway. Light grey fields are intermediate particles (PIP3) or pathways (VEGF). The box lists the processes influenced by selected pathways. Abbreviations: TLR, Toll-like receptor; PAMPs, pathogen-associated molecular patterns; BCR, B cell receptor; ECM, extracellular matrix; PIP3, phosphatidylinositol-3,4,5-triphosphate.

The comparison of locally expressed miRNAs pointed to many disease-specific miRNAs, with only two (miR-221-3p and miR-155-5p) reported in all three described conditions (Figure 2a). However, the analysis of genes targeted by these miRNA groups showed many shared targets (Figure 2b). An in silico functional analysis for more than four thousand common target genes indicated that the main pathways are crucial for intracellular signal transduction (PI3K-Akt, MAPK and RAS) (*p* < 0.05, FDR < 0.05, fold enrichment > 1.5). Furthermore, the KEGG database showed the functional network between these three pathways (Figure 3). PI3K-Akt, MAPK and RAS have been widely studied in the context of respiratory diseases [101,102,103]. No pathway for disease-specific miRNA sets (with excluded shared miRNAs) met the in silico functional analysis requirements. In summary, the investigated miRNAs are present in many respiratory conditions and might share fundamental signal transduction pathways. These similarities also reduce the probability of finding unique miRNA biomarkers of the disease.

Interestingly, we found a pathway meeting the analysis criteria for targets of miRNAs significantly upregulated in CF. These targets were involved in endocytosis regulation (*p* < 0.05, FDR < 0.05, fold enrichment = 2.1), through, e.g., Rab5, GRK and ARF6. The Rab5 subfamily regulates early endocytosis as well as the maturation of phagosomes, which are important for the innate immune cell responses [104].

From the two miRNAs shared/common in all diseases, only miR-221-3p showed the same direction of changes in all selected diseases (upregulation). miR-221 was previously described in the context of protein folding (CF), eosinophilia (asthma) and the progression of lung sarcoidosis (targeting, e.g., VEGF). An in silico verification pointed to its strong connection to the PI3K-Akt signalling pathway, which is functionally linked to the VEGF pathway (Figure 3). This is in line with the findings of Kiszałkiewicz et al. in pulmonary sarcoidosis [94]. Interestingly, there are ongoing studies on the use of VEGF and PI3K/AKT inhibitors in treating ILD, specifically in IPF [101,105,106]. miR-155 was also present in all three diseases, but it was reported as being upregulated in CF and ILD while being downregulated in asthma. This may be related to the differences in the course of the described diseases, since CF and ILD are characterised by fibrotic processes. At the same time, asthma is a strictly inflammatory condition. These two miRNAs share involvement in the, e.g., Ras and MAPK pathways. However, miR-155 explicitly targets genes involved in immunology response, such as TGF-β and T receptor signalling pathways, as well as the mTOR signalling pathway (*p* < 0.05, FDR < 0.05, fold enrichment from 2.8 to 3.4).

The presented analysis demonstrates the complexity and interdependence of multiple miRNAs that may interact in parallel with signalling pathways crucial to the progression of respiratory diseases.

## 6. Conclusions and Perspectives

Wide-range profiling of ncRNA expression may be limited due to the source material. Samples from the lungs might be contaminated with bacterial or fungal material from the lower respiratory tract or even oral flora, which is one of the major impediments. Since pathogenic DNA is also present in extracellular bacterial material originating from biofilms or dead cells, it might influence BALF or sputum RNA quality [107,108].

EBC is collected by the least-invasive method of all the biomaterials described above. However, it is characterised by a very low detection limit for expression analysis because of its considerable dilution [14]. The ncRNA expression profile is usually studied in either BALF or BB due to the abundance of nucleic acids. However, the increasing sensitivity of detection methods and analysis of free- and EV-transported nucleic acids will enable a better understanding of ncRNAs in other bodily fluids. A summary of the differences between the biosample types is shown in Table 4.

Despite severe qualitative and quantitative limitations, it is evident that all the described materials enable an understanding of epigenetic mechanisms involved in lung diseases. Non-coding RNAs can thus be studied both in cellular material and EVs using minimally invasive techniques and can be relatively easily accessible across age groups. Significant technical limitations include difficulties in refining the isolation protocol from body fluids, adjusting endogenous controls to normalise the results and a lack of the best biological samples to use for a particular disease. The present review clearly shows that ncRNAs are worth investigating in broader biosamples and disease perspectives due to their pleiotropic mode of action.

## Figures and Tables

**Table 4:** Comparison of selected biosamples collected from the respiratory tract. Described criteria relate to collection procedure and RNA quality/quantity.

	EBC	SPUTUM	BALF/BB
Invasiveness	None	Low (higher with induction)	High
Risk of bacterial contamination	Medium	High	Medium/high (BALF)
RNA abundance	Very low	Low/medium	High
Equipment and staff	Dedicated collection, devices and trained personnel	Collection pan and trained personnel (induction)	Specialised equipment (bronchoscope) and highly trained personnel
Time of sample collection	10–30 min	Depending on the expectoration capacity	Around 2 h

## Data Availability

No new data were created or analyzed in this study. Data sharing is not applicable to this article.
